# GABA deficiency in NF1: A multimodal [^11^C]-flumazenil and spectroscopy study

**DOI:** 10.1212/WNL.0000000000003044

**Published:** 2016-08-30

**Authors:** Inês R. Violante, Miguel Patricio, Inês Bernardino, José Rebola, Antero J. Abrunhosa, Nuno Ferreira, Miguel Castelo-Branco

**Affiliations:** From the Institute for Biomedical Imaging and Life Sciences, Faculty of Medicine (I.R.V., M.P., I.B., J.R., M.C.-B.), Laboratory of Biostatistics and Medical Informatics, Faculty of Medicine (M.P., M.C.-B.), and Institute of Nuclear Sciences Applied to Health (A.J.A., N.F., M.C.-B.), University of Coimbra, Portugal; and Division of Brain Sciences (I.R.V.), Department of Medicine, Hammersmith Hospital Campus, Imperial College London, UK.

## Abstract

**Objective:**

To provide a comprehensive investigation of the γ-aminobutyric acid (GABA) system in patients with neurofibromatosis type 1 (NF1) that allows understanding the nature of the GABA imbalance in humans at pre- and postsynaptic levels.

**Methods:**

In this cross-sectional study, we employed multimodal imaging and spectroscopy measures to investigate GABA type A (GABA_A_) receptor binding, using [^11^C]-flumazenil PET, and GABA concentration, using magnetic resonance spectroscopy (MRS). Fourteen adult patients with NF1 and 13 matched controls were included in the study. MRS was performed in the occipital cortex and in a frontal region centered in the functionally localized frontal eye fields. PET and MRS acquisitions were performed in the same day.

**Results:**

Patients with NF1 have reduced concentration of GABA+ in the occipital cortex (*p* = 0.004) and frontal eye fields (*p* = 0.026). PET results showed decreased binding of GABA_A_ receptors in patients in the parieto-occipital cortex, midbrain, and thalamus, which are not explained by decreased gray matter levels.

**Conclusions:**

Abnormalities in the GABA system in NF1 involve both GABA concentration and GABA_A_ receptor density suggestive of neurodevelopmental synaptopathy with both pre- and postsynaptic involvement.

Neurofibromatosis type 1 (NF1) is a neurodevelopmental monogenetic disorder, characterized by multisystemic symptoms, including increased incidence of cognitive deficits (50%–70%)^1^ and predisposition for tumor development.^2^ Cognitive impairments occur even in the absence of visible brain alterations, suggesting the existence of pathophysiologic abnormalities at a finer scale.

One hypothesis to explain the cognitive and behavioral profile is based on neurochemical imbalance in the γ-aminobutyric acid (GABA) system. Evidence for it includes (1) cellular and molecular studies in the *Nf1*^+/−^ model, which established a connection between RAS regulation by the *NF1* gene, GABAergic signaling, and learning deficits^3,4^; and (2) in vivo magnetic resonance spectroscopy (MRS) studies in children with NF1 showing reduced GABA levels.^5,6^

Moreover, it is possible that GABAergic alterations influence the distribution or binding affinity of GABA receptors, consistent with the “GABA shift.”^7^ This model postulates that increased GABA levels enhance the affinity of GABA type A (GABA_A_) receptors for benzodiazepine ligands. The directionality of changes in disease is, however, difficult to predict. On one hand, in homeostatic systems, the natural prediction is that, to maintain efficient neurotransmission, low GABA levels lead to increasing numbers of GABA receptors. On the other hand, reduced GABA concentration in patients could reflect a compensatory mechanism to counterbalance pre- and postsynaptically the effects of increased GABA release caused by reduced neurofibromin expression.^4^ GABA metabolism can be downregulated as a response to increased GABAergic neurotransmission, thus limiting GABA availability for packaging and release.^8^ If this were the case, we expect observing low GABA levels accompanied by a reduction in the number of GABA receptors. To further characterize the GABA system in adults with NF1, we studied the distribution of GABA_A_ receptors using [^11^C]-flumazenil PET, and GABA concentration using MRS.

## METHODS

### Participants.

We performed a cross-sectional study in 14 adult patients with NF1, diagnosed by the NIH criteria,^9^ and 14 healthy controls, matched for age, sex, and educational level. All participants were right-handed.

Exclusion criteria included psychiatric disorder, neurologic illness affecting brain function other than NF1, IQ <75, epilepsy, traumatic brain injury, or a clinically significant intracranial abnormality detected on MRI. T2 hyperintensities (commonly found in NF1) were not considered an exclusion criterion for patients. However, if present within the voxels prescribed for spectroscopy, the correspondent spectrum was not included. Our final cohort comprised 14 patients with NF1 (mean age 34.7 ± 7.1 years, 4 males) and 13 matched controls (mean age 36 ± 8.9 years, 4 males) (table 1).

**Table 1:**
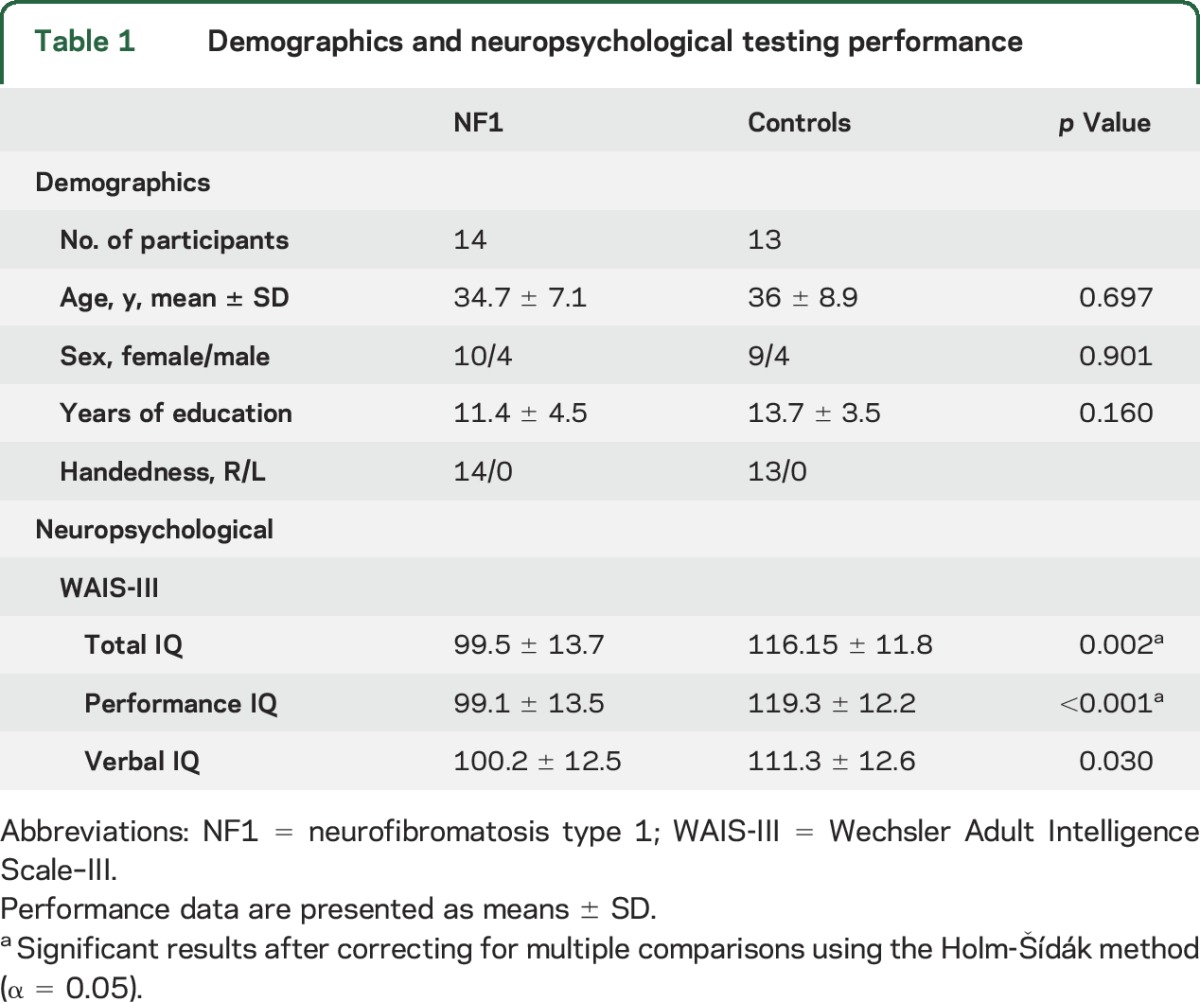
Demographics and neuropsychological testing performance

None of the participants were taking medication. Three patients reported taking antidepressants in the past (for periods ranging between 6 months to 3 years), but none were medicated in the year before the study. One control was medicated with antidepressants and stopped the medication a month before taking part in the study. None of the participants were ever medicated with anticonvulsants. Participants were instructed to withhold from drinking alcohol 24 hours before testing and from smoking or drinking coffee on the day of testing. All participants scored below the cutoff (<8) defined for hazardous and harmful alcohol use in the AUDIT (Alcohol Use Disorders Identification Test).^10,11^ Groups did not significantly differ in their scores (NF1: 2.07 ± 1.69; controls: 2.85 ± 2.54; *t*_25_ = 0.939, *p* = 0.36).

### Neuropsychological testing.

IQ was assessed using the Portuguese version of the Wechsler Adult Intelligence Scale–III.^12^ Patients with NF1 had IQ values in the normal range but significantly lower than controls (independent-samples *t* tests; Total IQ, *p* = 0.002; Performance IQ, *p* < 0.001; Verbal IQ, *p* = 0.030).

### Imaging.

Participants underwent all imaging acquisitions in the same day. PET and MRI order was counterbalanced. MRS was performed in the occipital cortex, to investigate whether GABA deficits persist into adulthood, and the frontal eye fields (FEFs). The latter was chosen because of its numerous cortical inhibitory circuits and because its location is easily identified using a functional localizer.

#### MRI and MRS acquisitions.

**Figure 1:**
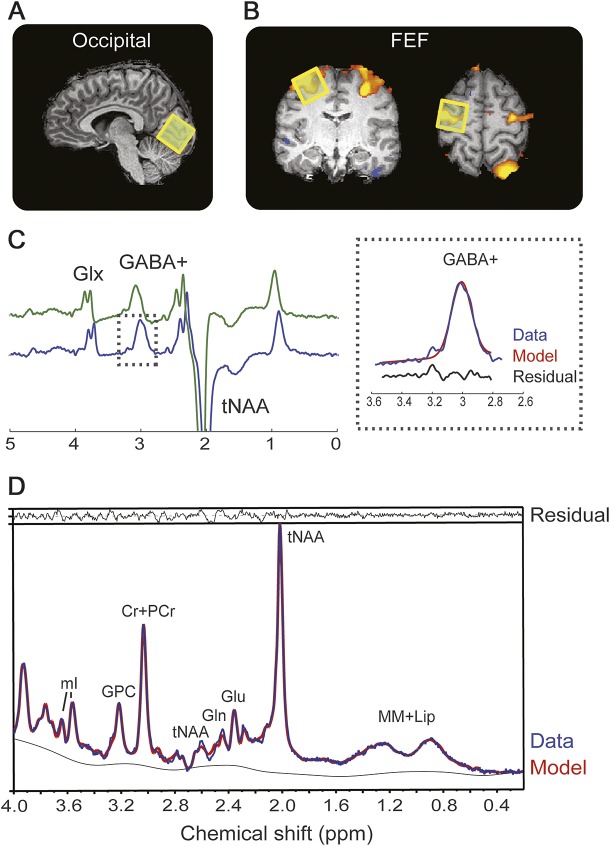
Magnetic resonance spectroscopy measurements Localization of the magnetic resonance spectroscopy voxel (yellow square) in the visual cortex (A) and FEF as localized with a functional localizer (B). (C) Edited magnetic resonance spectroscopy spectrum from a representative participant showing clearly resolved peaks for GABA+ and glutamine + glutamate (Glx). The inset on the right shows the fit output for the GABA+ signal. MEGA-PRESS spectra were processed using the Gannet 2.0 toolkit, the green line shows the raw GABA data, the blue line the postphase and frequency aligned GABA data, and the black line is the residual difference between the experimental data and the curve fit. (D) Single-voxel–localized PRESS spectrum (blue line) from a representative participant with spectral fits (red line) and the residual difference between the experimental data and the curve fit (black line) determined using LCModel. Cr = creatine; FEF = frontal eye field; GABA = γ-aminobutyric acid; Gln = glutamine; Glu = glutamate; GPC = glycerophosphocholine; mI = *myo*-inositol; PCr = phosphocreatine; tNAA = total *N*-acetylaspartate (NAA [*N*-acetylaspartate] + NAAG [*N*-acetylaspartylglutamine]).

Scanning was performed using a 3T Tim Trio (Siemens, Erlangen, Germany) with a 12-channel head coil. Acquisitions were as follows: (1) T1-weighted magnetization-prepared rapid-acquisition gradient echo for voxel placement and volumetric measurements; (2) T2-weighted fluid-attenuated inversion recovery to identify T2 hyperintensities; (3) functional localizer for the FEF using single-shot echo planar imaging. Participants performed an oculomotor task as described in the e-Methods at Neurology.org. Individual brain activation was examined using Neuro3D (Siemens) to allow immediate placement of the MRS voxel. (4) 2xGABA-edited spectra, MEGA-PRESS method.^13^ The occipital voxel was positioned within the occipital cortex with its lower face aligned with the cerebellar tentorium (figure 1A and figure e-1 for position across all participants). The FEF voxel was positioned based on the blood oxygen level–dependent (BOLD) activation elicited by the functional localizer (figure 1B and figure e-1 for position across all participants). In 2 NF1 participants, the voxel was placed according to anatomical landmarks (junction of precentral sulcus and superior frontal sulcus) because of insufficient BOLD activation. All voxels were placed on the right FEF with the exception of 2 participants who showed a lateralization of the FEF activation to the left hemisphere (2 patients with NF1). (5) ^1^H-PRESS in the occipital and FEF positions. Detailed scan measures can be found in the e-Methods.

#### MRI volumetric analyses.

T1-weighted images were segmented using the VBM8 toolbox (http://dbm.neuro.uni-jena.de/vbm/) in SPM8 (http://www.fil.ion.ucl.ac.uk/spm) to determine the relative proportions of gray matter (GM), white matter (WM), and CSF. This was used to determine tissue proportions in the spectroscopic voxels using Matlab7 (The MathWorks Inc., Natick, MA) and to perform voxel-based morphometry (e-Methods). The latter was used to investigate the potential confound that group differences in [^11^C]-flumazenil binding could partially arise from counterpart differences in GM volume.^14^

#### fMRI analyses.

Data processing and analysis were performed in BrainVoyager QX2.3 (Brain Innovation, Maastricht, the Netherlands). Preprocessing included slice time correction, linear trend removal, temporal high-pass filtering (3 cycles per run), motion correction, and spatial smoothing (full width at half maximum 5-mm gaussian kernel). No participant had within-run movements >3 mm. Predictors for the general linear model were built by convolving a boxcar time course with a 2-gamma function. BOLD signal peak amplitude (% signal change) for the pro- and antisaccade contrasts, at the peak voxel, were extracted for each participant in the FEF and occipital regions.

#### MRS analyses.

##### MEGA-PRESS.

The signal detected at 3.02 ppm is known to contain contributions from both macromolecules and homocarnosine,^15^ and is therefore referred to as GABA+. Quantification of GABA+ (figure 1B) was performed using the Gannet 2.0 toolkit^16^ to yield a GABA+/H_2_O ratio (institutional units). Only spectra with relative fit error <15% were included. Based on this criterion, the FEF spectra from 2 controls and 4 NF1s were excluded. For one control, the FEF spectrum was not acquired because of technical difficulties. Therefore, we included data from the FEFs of 10 controls and 9 patients. For the occipital voxel, we included data from 12 controls and 14 patients as one control was identified as an outlier (upper fence/hinge) and excluded from the analysis.

To account for differences in voxel tissue composition, the voxel fraction of WM (f_WM_) and GM (f_GM_) was used to normalize the GABA+ concentration according to: GABA+_corr_ = GABA+/(f_WM_ + f_GM_).

##### PRESS.

LCModel software (version 6.3) was used for metabolite quantification applying the internal water reference method, accounting for different water content in GM, WM, and CSF.^17^ Only metabolites with Cramér–Rao bounds <20% were considered. Concentrations of *N*-acetylaspartate (NAA) plus *N*-acetylaspartylglutamine (NAAG), creatine plus phosphocreatine, glycerophosphocholine, *myo*-inositol, glutamate, and glutamine were included for analysis. Concentrations in millimole units were calculated for all metabolites and results are presented in institutional units.

#### [^11^C]-Flumazenil PET.

A Philips Gemini GXL scanner (Philips Medical Systems, Best, the Netherlands) was used to acquire a CT scan and 26 sequential 3-dimensional PET frames of the entire brain (90 slices, 2-mm slice sampling), over a period of 60 minutes, after the bolus injection of 584.6 ± 25.9 MBq [^11^C]-flumazenil. PET data were reconstructed using a LOR-RAMLA algorithm. Voxel-by-voxel binding potential (BP) was calculated from dynamic PET images using the basis function implementation of the Simplified Reference Tissue Model, with the pons as reference.^18,19^ Several studies showed that using the pons activity as a reference is reliable and highly correlated with the BP values estimated by the arterial sampling method.^20^ Moreover, to ensure that systematic differences in the pons time-activity curves did not affect the results, we compared standard uptake values in the pons between the 2 groups (independent-samples, *t*_25_ = 0.370, *p* = 0.714).

PET data were normalized to Montreal Neurological Institute (MNI) space after rigidly coregistering the individual PET scan to the participant anatomical T1 scan in SPM8. The latter were segmented using the unified segmentation method^21^ in order to extract the transformation map into MNI space. This was then used to nonlinearly warp the PET scans. The resulting normalized images were smoothed using an isotropic gaussian kernel (12 mm full width at half maximum).

We performed exploratory whole-brain and region-of-interest (ROI)-based analyses. Whole-brain group differences were evaluated in SPM8 using independent-samples *t* test. [^11^C]-Flumazenil BP differences were considered statistically significant at peak height threshold of uncorrected *p* < 0.005 with cluster size greater than 230 contiguous voxels, which corresponds to a threshold of *p* < 0.05 corrected by cluster level for multiple comparisons as estimated by 10,000 Monte Carlo simulations in 3dClustSim (AFNI, http://afni.nimh.nih.gov/afni/). ROI-based analyses were performed in regions used for spectroscopy. The mean BP at these voxels was determined with the objective of investigating the relation between GABA concentration and GABA_A_ BP.

### Statistical analyses.

Data were transferred to SPSS version 21 (IBM Corp., Armonk, NY) for analysis. All dependent variables were tested for normality of distribution using the Shapiro-Wilk test and for outliers using the Tukey method. All variables were normally distributed.

Group differences were evaluated using independent-samples *t* tests and corrected for multiple comparisons using the Holm-Šídák method (α = 0.05). ROI analyses were performed using independent-samples *t* test and Pearson correlations (robustness estimated using bootstrapping, 10,000 iterations, to provide 95% confidence intervals). Effect sizes were calculated using Cohen *d*. Variance was similar between the groups as tested using the Levene test of homogeneity of variances.

### Standard protocol approvals, registrations, and patient consents.

Study procedures were reviewed and approved by the Ethics Commission of the Faculty of Medicine of the University of Coimbra. Participants provided written informed consent.

## RESULTS

### MRS results.

**Figure 2:**
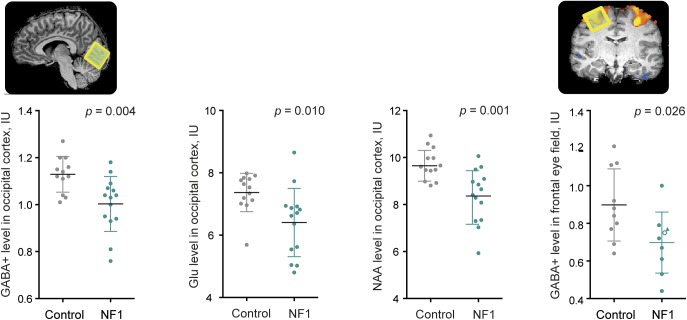
Magnetic resonance spectroscopy alterations in patients with NF1 Metabolites showing differences between patients with NF1 (green) and controls (gray) in the occipital cortex and the FEF. The triangle shape indicates a patient with NF1 in whom GABA+ was measured in the left hemisphere and the white-filled circle represents a patient in whom the FEF location was determined using anatomical landmarks. Graphs depict individual values, mean, and SD. FEF = frontal eye field; GABA = γ-aminobutyric acid; Glu = glutamate; NAA = *N*-acetylaspartate; NF1 = neurofibromatosis type 1.

Patients with NF1 displayed decreased GABA+ levels, of on average 11.5%, in the occipital cortex (*t*_24_ = 3.172, *p* = 0.004, *d* = 1.14) and 22% in the FEF (*t*_17_ = 2.443, *p* = 0.026, *d* = 1.27) (figure 2). In addition, patients showed lower levels of NAA + NAAG (*t*_25_ = 3.361, *p* = 0.002, *d* = 1.31; 12% reduction) and glutamate (*t*_25_ = 2.787, *p* = 0.01, *d* = 1.08; 10% reduction) in the occipital cortex (figure 2). A full summary of the spectroscopy results can be found in table e-1.

### PET results.

**Figure 3:**
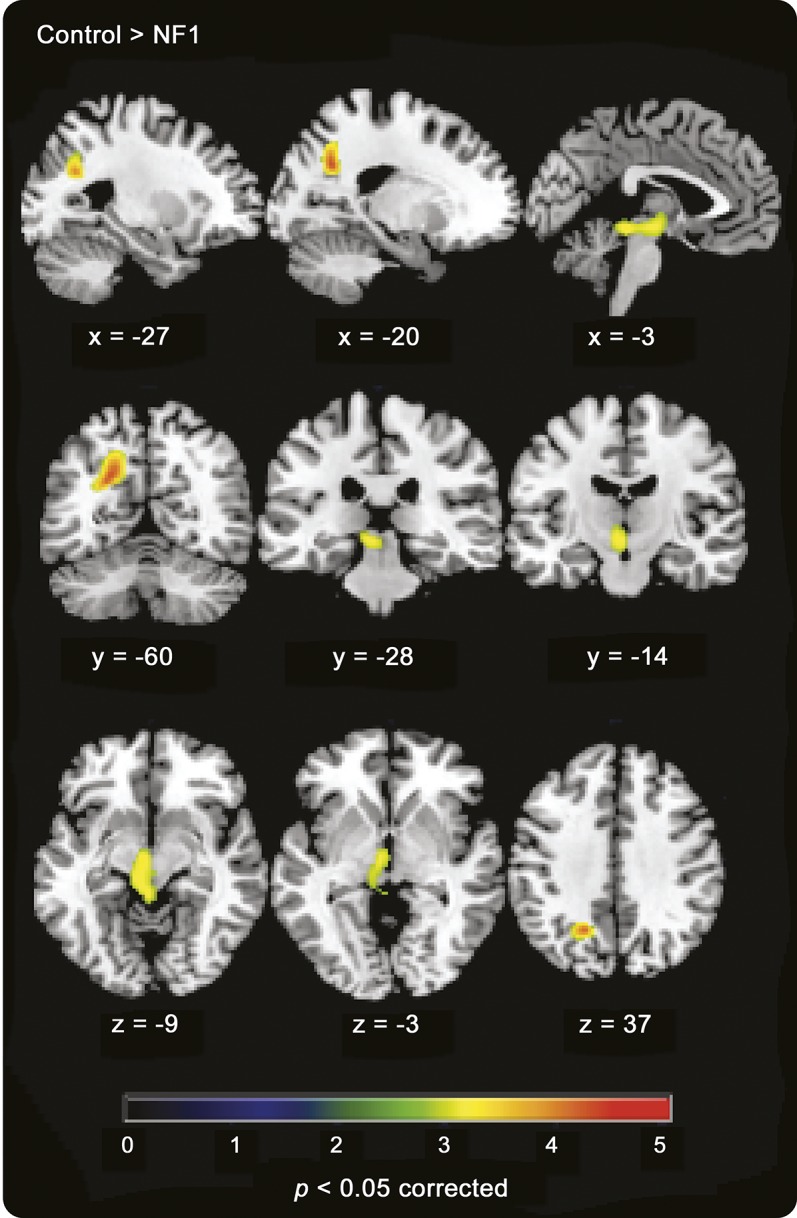
[^11^C]-Flumazenil PET binding differences between patients with NF1 and controls Areas showing decreased [^11^C]-flumazenil binding in patients (contrast control > NF1, *p* < 0.05 corrected). NF1 = neurofibromatosis type 1.

Whole-brain voxel-based analyses indicated that patients with NF1 have decreased [^11^C]-flumazenil BP in a left parieto-occipital region that encompasses the precuneus (MNI coordinates_Peak voxel_: [−22 −60 30]; *z* score = 3.65, k = 381, *p* < 0.05 corrected, *d* = 1.89) and in a region that includes the left midbrain and thalamus (MNI coordinates_Peak voxel_: [−2 −34 −10]; *z* score = 2.95, k = 447, *p* < 0.05 corrected, *d* = 1.64), whereas no brain regions displayed increased BP in patients (figure 3). Moreover, VBM showed that decreased [^11^C]-flumazenil BP in patients is not explained by decreased GM content, as decreased GM and decreased BP in patients do not colocalize (figure e-2). Moreover, to ensure that PET results were not hindered by concomitant abnormalities in GM, we performed correlation analyses between GM density and BP in the regions showing decreased PET BP. No correlation was observed for patients or controls (e-Methods), confirming that decreased BP in patients is not explained by GM alterations.

ROI-based analysis for the occipital and FEF voxels showed no statistically significant group differences in BP (occipital: *t*_25_ = 0.519, *p* = 0.608, *d* = 0.20; FEF: *t*_24_ = 0.936, *p* = 0.359, *d* = 0.37).

### Cross-modality correlations.

**Figure 4:**
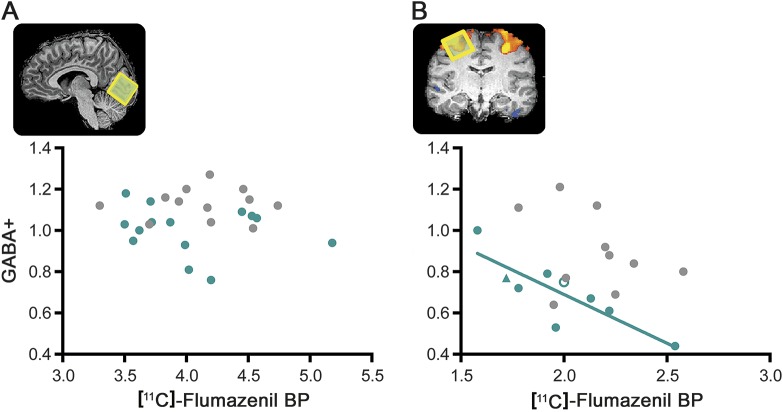
Cross-modality correlations between magnetic resonance spectroscopy and PET (A) Results for the occipital voxel and (B) for the FEF voxel, for patients with NF1 (green) and controls (gray). The triangle shape indicates a patient with NF1 in whom measurements were performed in the left hemisphere and the white-filled circle represents a patient in whom the FEF location was determined using anatomical landmarks. In patients with NF1, the concentration of GABA+ was negatively correlated with the density of GABA type A receptors in the FEF (*r* = −0.842, *p* = 0.004, n = 9, 95% confidence interval = −1.000 to −0.368 calculated from 10,000 bootstrap samples). BP = binding potential; FEF = frontal eye field; GABA = γ-aminobutyric acid; NF1 = neurofibromatosis type 1.

The relationship between the density of GABA_A_ receptors and the concentration of GABA was examined in occipital and FEF voxels. In patients, the concentration of GABA+ was negatively correlated with the density of GABA_A_ receptors in the FEF (*r* = −0.842, *p* = 0.004, n = 9, 95% confidence interval = −1.000 to −0.368) but not in the occipital cortex (*r* = −0.211, *p* = 0.469, n = 14) (figure 4). In controls, we did not find any significant correlation, neither in the FEF (*r* = −0.342, *p* = 0.334, n = 10) nor in the occipital cortex (*r* = 0.013, *p* = 0.969, n = 12). Yet, given the correlation values in the FEF and the low sample size, it is possible that the absence of significance is attributable to a type II error.

Finally, we examined whether individual peak BOLD amplitudes elicited by the oculomotor task were related with GABA concentration or GABA_A_ BP. There was no significant correlation between either GABA_A_ BP or GABA+ in neither the FEF nor occipital cortex for any of the groups.

## DISCUSSION

We report a comprehensive study of the GABA system in patients with NF1 that investigated cortical GABA concentration using MRS and GABA_A_ receptor density using PET imaging. Our results indicate that patients with NF1 have decreased GABA+ levels and decreased GABA_A_ receptors, corroborating our hypothesis that alterations in the GABA system extend beyond reduced GABA concentration.

Spectroscopy results showed that reduced GABA levels in the occipital cortex persist into adulthood, confirming our initial findings in children with NF1,^5,6^ and that this pattern is additionally observed in a frontal region centered in the FEF. Altogether, the observation of reduced GABA levels in several cortical locations (medial frontal cortex,^5^ occipital, and FEF) suggests a ubiquitous cortical dysfunction. Moreover, in the occipital cortex, GABA reductions were accompanied by lower glutamate and NAA + NAAG concentrations. This reduction in NAA is in agreement with earlier reports in subcortical structures^22,23^ and WM.^24^ The fact that both glutamate and NAA are altered might be explained by the tight link between these metabolites; they are interconnected through a series of biochemical reactions, mainly the tricarboxylic acid cycle and glutamate–glutamine cycle.^25^ It is therefore likely that reduced levels of GABA, glutamate, and NAA are linked and contributing to maintaining low levels of GABA available for neurotransmission. Furthermore, our results are in agreement with recent findings in the *Nf1*^+/−^ model showing GABAergic and glutamatergic alterations.^26^

GABA_A_ receptor density was also found to be reduced in patients in discrete locations: parieto-occipital cortex, midbrain, and thalamus. Alterations in the parieto-occipital cortex are in agreement with functional^27,28^ and anatomical^29,30^ abnormalities reported in NF1. Likewise, thalamic metabolic^31–33^ and volumetric alterations^30^ have been previously reported. Both the thalamus and midbrain are important relay stations of sensory inputs to the cortex. GABAergic neurons in the thalamus are involved in the generation of synchronized activity in thalamocortical networks,^34^ a critical process for cognitive functions. In the midbrain, GABAergic neurons are involved in controlling the firing pattern of dopaminergic neurons.^35^

Of note, PET results were not hindered by concomitant abnormalities in GM. It is advisable that studies investigating differences in GABA_A_ density using flumazenil perform GM comparisons independently, as the relationship between flumazenil BP and GM volume^36^ or cortical thickness^14^ is not homogeneous across brain regions. VBM results showed that decreased BP and decreased GM density do not colocalize and are not correlated, indicating that decreased GM does not explain decreased density of GABA_A_ receptors. Of note, we found that increased GM volume and decreased [^11^C]-flumazenil BP in patients colocalizes, in a region including left midbrain and left thalamus. Here, GM volume is enlarged in the absence of a parallel increase in inhibitory neurons. This could either be an indication of increased populations of other neuronal types, namely, dopaminergic and glutamatergic, or that the population of inhibitory neurons preferentially expresses other types of GABA receptors. Studies with PET ligands to other receptors or postmortem studies could disentangle this hypothesis.

Our findings revealed a complex pattern of alterations in the GABA system in NF1, with both GABA+ and GABA_A_ reductions, yet in different locations. In addition, a negative correlation between GABA+ and the density of GABA_A_ receptors was observed in the FEF of patients and not in the occipital cortex. The fact that this relationship was not observed in controls is an indication that natural variation is not tightly linked for these 2 variables.

It is important to note that MRS and PET are complementary techniques measuring different components of the GABA system and it is possible that different brain regions give primacy to different regulatory mechanisms. Studies measuring GABA+ in regions showing altered GABA_A_ binding could help disentangle the hypothesis that a reduction in both GABA levels and GABA_A_ receptors can occur as part of a regulatory mechanism to deal with the detrimental effects of increased GABAergic neurotransmission.

Finally, it is noteworthy that both GABA MRS and [^11^C]-flumazenil PET are subject to limitations. Particularly important to this study is the fact that MRS is not capable of distinguishing between metabolic and synaptic pools, and [^11^C]-flumazenil PET is not specific for the synaptic subtypes of the GABA_A_ benzodiazepine receptor, as it shows affinity to both synaptic and extrasynaptic receptor subtypes.^37^

Overall, we now have evidence of alterations occurring at multiple levels of the GABA pathway. Studies in animal models have shown presynaptic alterations that lead to increase GABA release,^4,26^ while our studies in humans show that the overall concentration of GABA is reduced as well as the expression of GABA_A_ receptors. Moreover, we showed that deficits in GABA levels do not ameliorate with age. Of note, abnormalities in the GABA system in NF1 are involved in relevant behavioral and cognitive domains including social learning,^26^ memory,^28^ and inhibitory control.^5^

In sum, our results point to abnormalities in the GABA system suggestive of synapthopathy, with both pre- and postsynaptic involvement.

However, given the discrete distribution of GABA_A_ receptor abnormalities, it is difficult to support that treatments with GABA_A_ antagonists, as suggested from the animal literature,^4^ would be useful to treat cognitive deficits in humans.

## Supplementary Material

Data Supplement

## GLOSSARY

BOLD: blood oxygen level–dependent
BP: binding potential
FEF: frontal eye field
GABA: γ-aminobutyric acid
GABA_A_: γ-aminobutyric acid type A
GM: gray matter
MNI: Montreal Neurological Institute
MRS: magnetic resonance spectroscopy
NAA: *N*-acetylaspartate
NAAG: *N*-acetylaspartylglutamine
NF1: neurofibromatosis type 1
ROI: region of interest
WM: white matter
