# Supplementary material for: GABA deficiency in NF1: A multimodal [11C]-flumazenil and spectroscopy study
Source: Neurology. 2016 Aug 30;87(9):897–904. doi: 10.1212/WNL.0000000000003044 (PMC5035153; doi:10.1212/WNL.0000000000003044)
Supplement: Data Supplement [file supp_87_9_897__index.html]

GABA deficiency in NF1 — Data Supplement 

# GABA deficiency in NF1

## Data Supplement

**Neurology® data supplements are not copyedited before publication. Published editorials and translations have been copyedited.  
 © 2016 American Academy of Neurology.  
  
 Files in this Data Supplement:**

- Data Supplement - PDF
