# Supplementary material for: GABA deficiency in NF1: A multimodal [11C]-flumazenil and spectroscopy study
Source: Neurology. 2016 Aug 30;87(9):897–904. doi: 10.1212/WNL.0000000000003044 (PMC5035153; doi:10.1212/WNL.0000000000003044)
Supplement: Data Supplement [file supp_WNL.0000000000003044_Violanteetal_SupplementaryMaterial.pdf]

Supplemental Material

GABA deficiency in NF1: a multimodal [^{11}C]-Flumazenil and spectroscopy study

Inês R. Violante, Miguel Patricio, Inês Bernardino, José Rebola, Antero J. Abrunhosa,
Nuno Ferreira, Miguel Castelo-Branco

Supplementary Methods

MRI and MRS acquisitions

Scanning was performed on a 3T Siemens TimTrio (Siemens), using a 12-channel head coil. Acquisitions: (i) T1-weighted MP-RAGE, 1 mm^3 isotropic voxel, repetition time (TR) 2530 ms, echo time (TE) 3.42 ms, inversion time (TI) 1100 ms, flip angle (FA) 7°, field of view (FOV) 256 x 256 mm, 256 x 256 matrix, 176 slices, GRAPPA = 2; (ii) T2-weighted FLAIR to identify T2-hyperintensities, 1 mm^3 isotropic voxel, TR 5 s, TE 388 ms, TI 1.8 s, FOV 250x250 mm, 256x256 matrix, 160 slices, GRAPPA = 2; (iii) functional localizer for the frontal eye fields (FEF) using single-shot EPI, 3.6 mm^3 isotropic voxel, TR 1.5 s, TE 30 ms, FA 76°, FOV 230x230 mm, 64 x 64 matrix, 28 slices, GRAPPA = 2; (iv) 2xGABA-edited spectra using the MEGA-PRESS method[1, 2], 3 cm^3 isotropic voxel, TE 68 ms, TR 1.5 s, 196 averages (occipital) and 264 averages (FEF), 1024 data points. The occipital voxel was positioned within the occipital cortex with its lower face aligned with the cerebellar tentorium (Figure e-1A). The FEF voxel was positioned based on the BOLD activation elicited by the functional localizer. In two NF1 participants the voxel was placed according to anatomical landmarks (junction of precentral sulcus and superior frontal sulcus), due to insufficient BOLD activation. All voxels were placed on the right FEF with the exception of two participants that showed a lateralization of the FEF activation to the left hemisphere (two patients with NF1), (Figure e-1B). (v) ^{1}H-PRESS in the occipital and FEF positions, 3 cm^3 isotropic voxel, TE 35 ms, TR 2 s, 46 averages, 1024 data points.

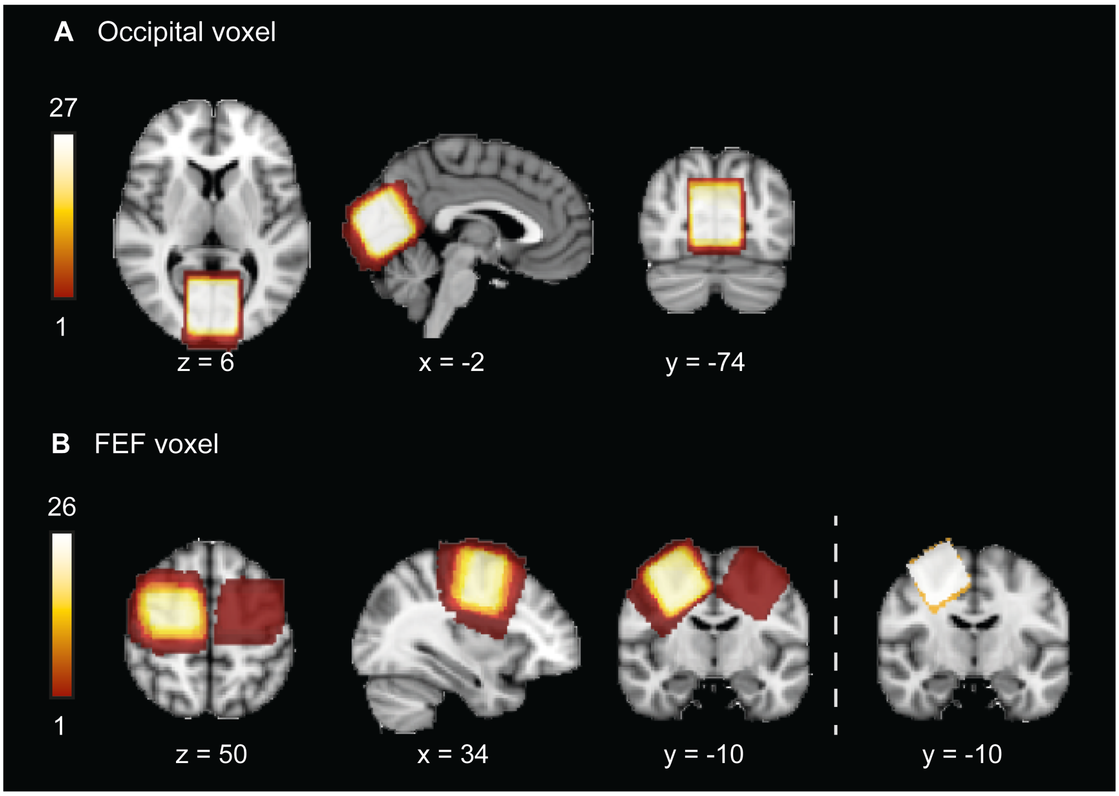

Figure e-1. MRS voxel positions.

A. Overlay of the occipital cortex voxel in all subjects (The color bar indicates the number of subjects). **B.** Left panel: overlay of the FEF voxel in all subjects (color bar indicates the number of subjects). Right panel: overlay of the two MRS voxels positioned according to anatomical landmarks.

Images are in MNI space and radiological orientation.

Occulomotor task to localize the frontal eye fields (FEF)

Trials began with fixation on a central white dot (2° of visual angle) for 1500 ms, followed by presentation of a coloured dot for 1300 ms, which cued for a pro- (green dot) or anti-saccade (red dot). A subsequent gap of 200 ms occurred prior to target presentation for 100 ms (white square 4° of visual angle) at 6 or 7° to the left or right of fixation. Subjects had 1400 ms to perform a saccade to the location of the target on a pro-saccade trial, or to its mirror location on an anti-saccade trial. A total of 20 pro- and 20 anti-saccade trials were presented in a block design (18 s per block, 5 blocks per task) interleaved with 12 s rest blocks (fixation). We monitored eye movements using a camera placed on the mirror system (Avotec Real Eye 5721).

Voxel-based morphometry

It is important that studies investigating differences in $GABA_A$ density employing flumazenil perform grey matter comparisons independently as the relationship between flumazenil BP and grey matter volume[3] or cortical thickness[4] is not simple as their correlation is not homogeneous across brain regions.

To investigate the potential cofound that group differences in binding potential could partially arise from counterpart differences in grey matter (GM) volume, we performed voxel based morphometry, applying standard procedures implemented in the VBM8 toolbox (http://dbm.neuro.uni-jena.de/vbm/) in SPM8 (http://www.fil.ion.ucl.ac.uk/spm). This included, correction for bias-field inhomogeneity, registration using linear and nonlinear transformations and segmentation into GM, white matter (WM) and CSF images. Images were smoothed with a FWHM of 8 mm.

Group comparisons were performed using voxel-wise independent-samples t-tests in SPM and corrected for multiple comparisons using a peak height threshold of uncorrected $P < 0.005$ with cluster size greater than 1352 contiguous voxels as estimated by 10,000 Monte Carlo simulations in 3dClustSim (AFNI, http://afni.nimh.nih.gov/afni/). This corresponds to a threshold of $P < 0.05$ corrected for multiple comparisons.

Supplementary Results

Spectroscopy results

Supplemental Table e-1 Spectroscopy results

	Metabolite Level, Mean ± SD, IU									
	Occipital Cortex					Frontal Eye Field				
Metabolite	NF1	N	Controls	N	*P*	NF1	N	Controls	N	*P*
GABA+	1.00 ± 0.12	14	1.13 ± 0.08	12	**0.001***	0.70 ± 0.16	9	0.90 ± 0.19	10	**0.026***
Gln	3.58 ± 0.53	12	3.86 ± 0.82	13	0.323	2.53 ± 0.42	9	2.72 ± 0.44	10	0.369
Glu	6.40 ± 1.1	14	7.37 ± 0.62	13	**0.010***	5.11 ± 0.80	14	5.45 ± 0.86	12	0.296
GPC	1.02 ± 0.13	11	1.02 ± 0.10	12	0.882	1.31 ± 0.22	12	1.18 ± 0.19	10	0.169
mI	4.47 ± 0.50	14	4.52 ± 0.43	13	0.748	3.63 ± 0.57	14	3.47 ± 0.37	12	0.408
PCr+Cr	6.06 ± 0.78	14	6.37 ± 0.32	13	0.207	4.94 ± 0.40	14	4.82 ± 0.23	12	0.345
tNAA	9.44 ± 1.17	14	10.69 ± 0.67	13	**0.002***	8.27 ± 0.83	14	8.63 ± 1.02	12	0.361

Cr = creatine, Gln = glutamine, Glu = glutamate, GPC = glycerophosphocholine, mI = myo-Inositol, PCr = phosphocreatine, tNAA = total N-acetyl-aspartate (NAA (N-acetyl-aspartate) + NAAG (N-acetylaspartylglutamine)).

* Significant results after correcting for multiple comparisons using the Holm-Sidak method (alpha = 0.05). The N reflects the number of participants included for the statistical analysis. Only metabolites with Cramér–Rao bounds below 20% were considered.

Voxel-based morphometry results

VBM analyses showed that decreased [^{11}C]-Flumazenil binding potential (BP) in patients do not co-localize with the regions in which patients have decreased GM, Figure 2 (PET contrast Control > NF1 (magenta) and VBM contrast Control > NF1 (red)). Therefore, decreased density of $GABA_A$ receptors is not explained by decreased GM density.

In addition, VBM analyses identified significant decrease in GM volume in patients with NF1 in the precuneus and extending to the left paracentral lobule (MNI coordinates: [-6 -46 52]; t score 5.91, k = 1842, $P < 0.05$ corrected), Figure 2. The reverse contrast, NF1 > Control, showed increased GM volume in patients in a region encompassing the thalami and the vermis (MNI coordinates: [0 11 1]; t score 5.55, k = 1830, $P < 0.05$ corrected) and in another region that extends from the caudate to the subcallosal cortex and subgenual anterior cingulate cortex (MNI coordinates: [18 -24 -3]; t score 5.32, k = 1953, $P < 0.05$ corrected), Figure e-2.

Interestingly, increased GM volume and decreased flumazenil BP in patients co-localizes in a region including the left midbrain and left thalamus. In this region, patients have increased GM volume in the absence of a parallel increase in inhibitory neurons.

To ensure that PET results were not hindered by concomitant abnormalities in GM we performed correlation analyses between GM density and BP in the regions showing decreased PET BP. No correlation was observed for patients or controls in neither the thalamic/midbrain region [NF1: r = 0.185, P = 0.527, N=14; Controls: r = -0.153, P = 0.619, N=13] nor the parietal-occipital region [NF1: r = 0.479, P = 0.083, N=14; Controls: r = 0.500, P = 0.081, N=13], confirming that decreased BP in patients is not explained by GM alterations.

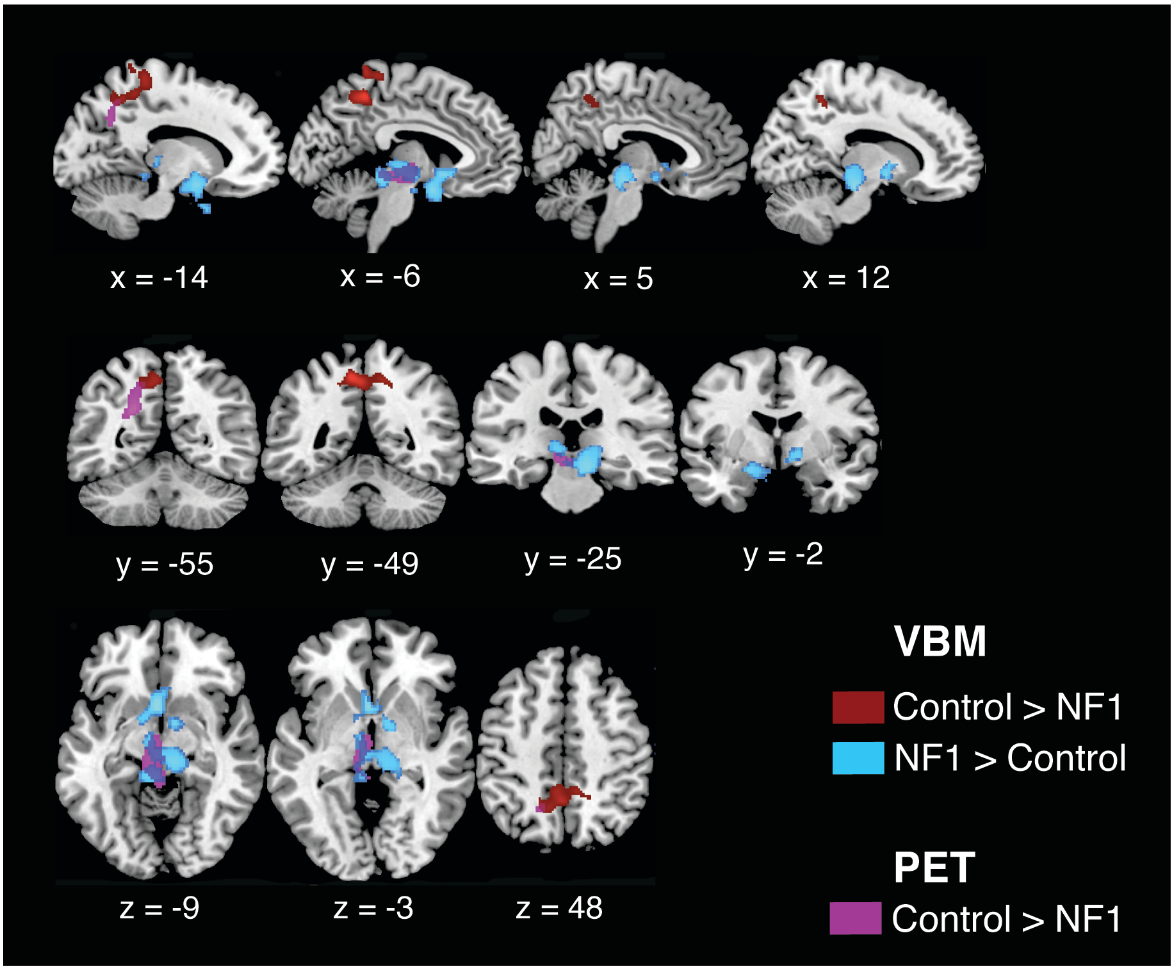

Figure e-2. Voxel-based morphometry (VBM) analysis between patients with NF1 and controls.

Areas of decreased grey matter volume in patients are displayed in red (Control > NF1) and areas of increased grey matter volume in patients are displayed in blue (NF1 > Control) at a level of $P < 0.05$ corrected (peak height threshold of uncorrected $P < 0.005$ with cluster size greater than 1352 contiguous voxels as estimated by 10,000 Monte

Carlo simulations). Regions of decreased grey matter volume in patients (red) do not co-localize with regions of decreased [^{11}C]-Flumazenil PET binding (pink).

References

1. Mescher M, Merkle H, Kirsch J, Garwood M, Gruetter R. Simultaneous in vivo spectral editing and water suppression. *NMR in biomedicine* 1998; **11**(6)**:** 266-272.

2. Edden RA, Barker PB. Spatial effects in the detection of gamma-aminobutyric acid: improved sensitivity at high fields using inner volume saturation. *Magn Reson Med* 2007; **58**(6)**:** 1276-1282.

3. Duncan NW, Gravel P, Wiebking C, Reader AJ, Northoff G. Grey matter density and GABAA binding potential show a positive linear relationship across cortical regions. *Neuroscience* 2013; **235:** 226-231.

4. la Fougere C, Grant S, Kostikov A, Schirrmacher R, Gravel P, Schipper HM *et al.* Where in-vivo imaging meets cytoarchitectonics: the relationship between cortical thickness and neuronal density measured with high-resolution [18F]flumazenil-PET. *Neuroimage* 2011; **56**(3)**:** 951-960.
